# Cholinesterases and Engineered Mutants for the Detection of Organophosphorus Pesticide Residues

**DOI:** 10.3390/s18124281

**Published:** 2018-12-05

**Authors:** Yu-Ling Xu, Feng-Ye Li, Ferdinand Ndikuryayo, Wen-Chao Yang, Hong-Mei Wang

**Affiliations:** 1 School of Chemical & Environmental Engineering, Wuhan Polytechnic University, Wuhan 430023, China; xyling619@whpu.edu.cn; 2 Key Laboratory of Pesticide & Chemical Biology of Ministry of Education, and International Joint Research Center for Intelligent Biosensor Technology and Health, and Chemical Biology Center, College of Chemistry, Central China Normal University, Wuhan 430079, China; fengye@mails.ccnu.edu.cn (F.-Y.L.); ndikuferdi@mails.ccnu.edu.cn (F.N.); 3 Hubei Key Laboratory of Wudang Local Chinese Medicine Research, School of Pharmacy, Hubei University of Medicine, Shiyan 442000, China

**Keywords:** cholinesterase, mutant, organophosphorus pesticides, sensor

## Abstract

Nowadays, pesticide residues constitute an increasing public health concern. Cholinesterases, acetylcholinesterase, and butyrylcholinesterase, are reported to be involved in detoxification processes owing to their capability of scavenging organophosphates and carbamates. Thus, these enzymes are targeted for the discovery of sensors aiming at detecting pesticide residues. In recent years, cholinesterase-based biosensors have attracted more and more attention in the detection of pesticides. Herein, this review describes the recent progress on the engineering of cholinesterases and the development of the corresponding sensors that could be used for the detection of organophosphorus pesticide residues.

## 1. Introduction

Pesticides play an important role in the agriculture field by increasing crop yields. Organophosphorus pesticides (OPs) are widely applied in crop protection due to their high efficacy and relatively low persistence in the environment. However, the widespread and long-term use of OPs has brought many consequences, such as the accumulation of pesticide residues in food, and the contamination of water and soil [1], resulting in public health and environmental concerns. For example, Geoffrey and Calvert [2] reported 1009 individuals from seven states that were diagnosed with acute occupational illness induced by pesticides between 1988 and 1999. Therefore, the early and accurate detection of pesticide residues is of great significance.

**Figure 1:**
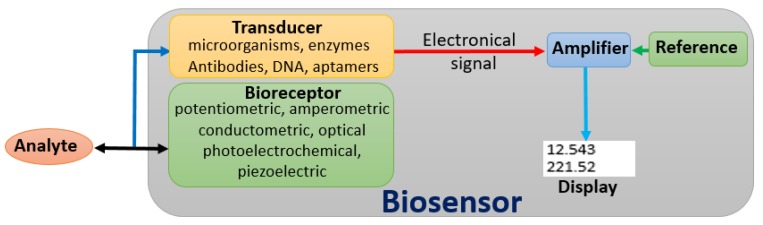
The basic components of biosensor. The black, blue and red arrows symbolize the chemical interaction, biochemical signal, and detectable electric signal, respectively.

To date, several methods for the detection of pesticide residues have been developed with the help of advanced technologies, including colorimetric assay [3], high-performance liquid chromatography (HPLC) [4], liquid chromatography (LC) [5], and mass spectrometer (MS) [6]. In general, most of these methods are usually employed to analyze pesticide residues in laboratories. Moreover, some conventional methods like MS and LC are not only laborious, but also require expensive equipment and highly trained technicians to meet the practical requirements [7]. Thus, there is an emerging need for an immediate, simple, and sensitive technique that could be used for the detection of pesticide residues. Biosensors have attracted the attention of many researchers worldwide due to their high selectivity and sensitivity for easy on-site and in situ determination. In addition, biosensors are sensitive to biological substances or analytes [8,9], and convert their concentration into detectable signals (Figure 1). Interestingly, biosensors are not only cost-effective, but also have great development potential.

In the animal body, a number of compounds, including acetylcholine (ACh) and butyrylcholine (BCh), achieve specific tasks through catalyzed reactions [10]. For example, acetylcholine (ACh) is hydrolyzed into acetic acid and choline (Ch), whereas butyrylcholine (BCh) is broken into butyric acid and Ch at neuron dendrites [11]. This hydrolysis process can be catalyzed by two major cholinesterases, i.e., acetylcholinesterase (AChE, EC 3.1.1.7) and butyrylcholinesterase (BChE, EC 3.1.1.8), which belong to serine hydrolase family. From presynaptic vesicles, AChE and BChE are released into the synaptic cleft, and then bind to the ACh receptor onto the postsynaptic membrane to transmit a signal to the next nerve cell [12]. 

Site-directed mutagenesis is commonly used to generate variant enzymes with improved characteristics such as increased selectivity, sensitivity, and stability [13]. Extensive mutagenesis studies have been carried out in order to find optimized enzymes. For example, PEG-F338A human AChE, compared with wild-type, was reported to be a prophylactic treatment against OP-poisoning [14], owing to its OP-bioscavenger capability. Moreover, the human BChE (*h*BChE) mutant G117H can inactivate many OP molecules [15]. 

## 2. Structure of AChE and BChE

**Figure 2:**
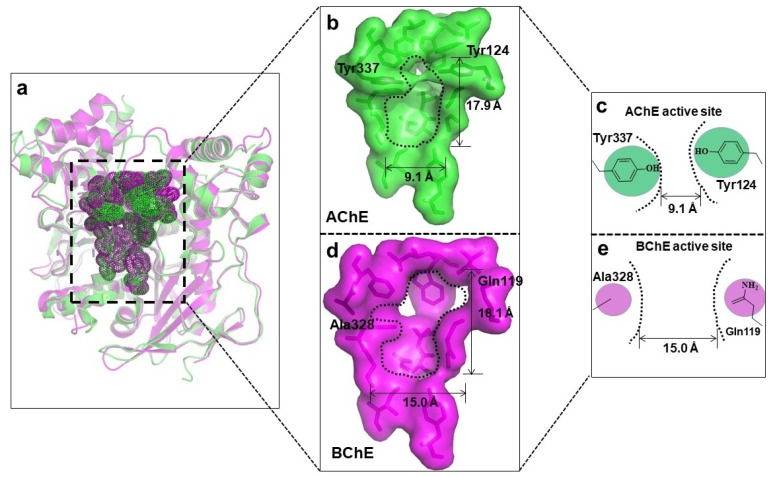
(**a**) The superimposition of the crystal structures of AChE (in green) and BChE (in purple). (**b**) The surface view of AChE and the active site is circled with a black dash line. (**c**) The size of the middle part of the AChE active site. (**d**) The sphere view of BChE and the active site is circled with a black dash line. (**e**) The size of the middle part of the BChE active site.

AChE is a vital member of the serine hydrolase family, and plays a key role in cholinergic transmission by catalyzing the rapid hydrolysis of neurotransmitter ACh into acetate and choline [16]. Therefore, AChE is an antidote to organic phosphorus and carbamate poisoning, as it is a serine hydrolyzing enzyme [17]. The basic structure of the AChE molecule is ellipsoidal, and possesses three binding sites, i.e., an active site (with catalytic anionic and esteratic subsites), aromatic gorge, and peripheral anionic site, where the inhibiting compounds interact. As shown in Figure 2, the overall crystal structures of *h*AChE and *h*BChE are very similar, but the sizes of their active sites are quite different. More specifically, the gorge of AChE is ~17.9 Å deep and 9.1 Å wide, while the gorge of BChE is ~18.1 Å deep and 15.1 Å wide. The middle part of the AChE gorge is much narrower than that of BChE, due to the presence of two aromatic residues (Tyr337 and Tyr124). As for AChE, it comprises 4 subsites. The first, peripheral site, is located at the entrance of the gorge, and controls the traffic of substrate and the products in the acylation region. The second subsite is the quaternary ammonium binding site, represented by Tyr70, Asp72, Trp84, and Phe330 residues. Both peripheral and ammonium binding sites are called peripheral aromatic sites, as they are composed of aromatic rings [18,19]. The other two subsites, the anionic (oxyanion hole) and catalytic subsites, are located much deeper in the gorge. 

BChE is composed of 574 residues and its gorge is made of 55 residues [20]. Although similar structures between *h*AChE and *h*BChE have been explained, the location of BChE and the response upon ligand binding differ significantly. Indeed, BChE is more open than AChE, and it does not form the dimer observed in previous structures of AChE from *Torpedo californica*, mouse, and human [21,22]. One of the most important differences is the residue composition of the gorge, which determines their specificity. For example, the replacement of Phe288 and Phe290 of *Tc*AChE by Leu286 and Val288 in BChE enables BChE to bind with the butyrate substrate, which is bulkier than acetyl [23]. Though AChE and BChE are related enzymes, chemical signaling pathways primarily rely on AChE as a key regulator [24], and hence, AChE is mostly considered an ideal target for the discovery of biosensors used for the detection of OPs. Thus, apart from a few reported BChE-based biosensors [19,25,26,27], the principle of most ChE-based biosensors relies on AChE [28]. 

## 3. Interactions of OPs Pesticides with ChEs

The inhibition of AChE by an organophosphorus ester takes place via a chemical reaction in which the serine hydroxyl moiety is phosphorylated in a manner analogous to the acetylation of AChE. In contrast to the acetylated enzyme, which rapidly breaks down to give acetic acid and the regenerated enzyme, the phosphorylated enzyme is highly stable and, in some cases, depending on the groups attached to the phosphorus atom (R and R’), is irreversibly inhibited [29]. Due to the blockage by a phosphoryl moiety, the serine hydroxyl group is no longer able to participate in the hydrolysis of ACh. 

**Figure 3:**
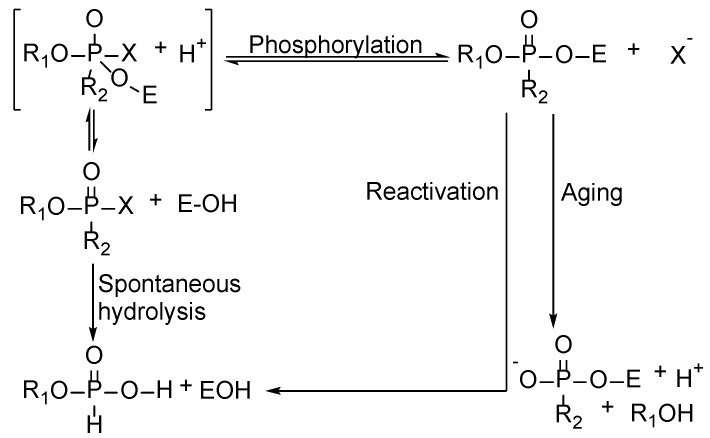
Mechanism of the inhibition of cholinesterase by OPs.

As shown in Figure 3, whereas one part of OPs undergoes spontaneous hydrolysis, another participates in the inhibition of AChE. The former reaction mainly comprises the phosphorylation of the enzyme via the formation of the enzyme-substrate complex. Phosphorylation of AChE or BChE by OPs results in an inactive enzyme that can no longer hydrolyze ACh, whose increased concentration in the junction may result in exhaustion [30]. After phosphorylation, segmental phosphorylated AChE can be reactivated and reproduce ChE. Meanwhile, a competitive process known as the aging of the phosphorylated AChE occurs. In this process, AChE is converted into its dealkylated form, while the alkoxyl group bonded to phosphor is replaced by the hydroxyl group [31]. 

## 4. Cholinesterase-Based Biosensors

The development of biosensors provides a new method for the rapid detection of pesticide residues. This technology has opened up new horizons for the development of new, rapid, and low-cost analytical methods that can be used for the detection of pesticide residues. As indicated in Figure 1, a biosensor can be defined as a self-contained device that integrates an immobilized biological element (bioreceptor) which recognizes the target molecule in complex mixtures (analyte), and a transduction element (transducer) that converts the biochemical signal resulting from the interaction of the analyte with the bioreceptor into an electronic signal [32]. After amplification, both electronic and reference signals are displayed on the screen. 

The specificity and sensitivity of biosensors mainly rely on the bioreceptor. Different types of bioreceptors can be used, such as whole cells or subcellular fragments of microorganisms, antibodies, DNA sequences, aptamers, or enzymes. But biosensors designed on the basis of enzymatic inhibition are particularly relevant, because pesticides are mainly designed on the principle of enzyme inhibition [33]. A transducer is another key component which can significantly determine the usefulness of a biosensor. Based on the type of the transducer, various ChE-based biosensors can be classified as potentiometric, amperometric, conductometric, photoelectrochemical, optical, and piezoelectric [34]. 

**Figure 4:**
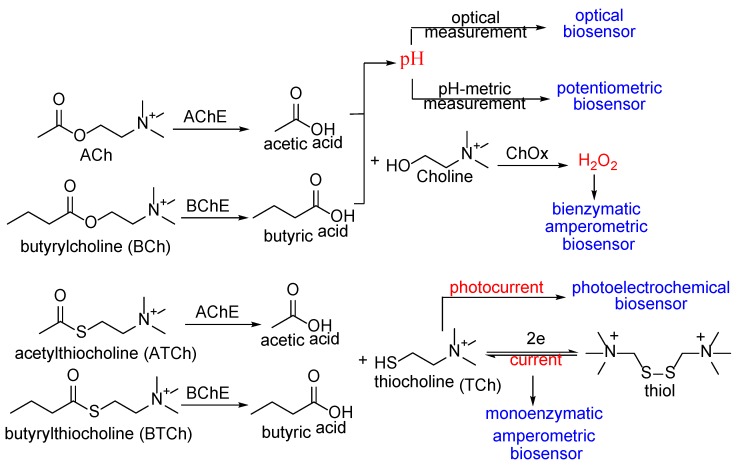
Principles of various types of biosensors.

Potentiometric, amperometric, conductometric biosensors are commonly known as electrochemical biosensors. Basic principles of various types of biosensors are shown in Figure 4. Potentiometric biosensors rely on the measurement of the variation in pH induced by the release of acetic or butyric acids in the medium [35]. These kinds of biosensors can be used for qualitative analysis using pH indicators [36]. Amperometric biosensors rely on the measurement of the current generated by oxidoreduction reaction. There are two types of amperometric biosensors: mono-enzymatic and bi-enzymatic. For the first type, the hydrolysis of ACh or butyrylcholine (BCh) yield thiocholine (TCh), which is oxidized to dithiol, thereby generating a current that is inversely proportional to the concentration of the inhibitor in the sample [37,38]. The hydrolysis of ACh and BCh, in the second type, generates Ch, which is then oxidized by choline oxidase (ChOx) to yield hydrogen peroxide (H_2_O_2_), that can be detected amperometrically [39]. For photoelectrochemical biosensors, thiocholine from acetylthiocholine (ATCh) hydrolysis generates a photocurrent that is proportional to the inhibitor concentration [40]. In the case of optical biosensors, the usual production of acetic or butyric acid provokes a change in pH that can be monitored optically [36]. 

Cholinesterase (ChE) biosensors have emerged as a sensitive and rapid technique for toxicity monitoring for environmental, agricultural, food, or military applications [41]. Early constructed ChE-based biosensors were mostly tested in the laboratory with promising properties. To evaluate the usefulness and transfer these devices to real-life and commercial applications were the main challenges. To overcome these challenges, research was mainly oriented towards the optimization of critical parameters such as enzyme stability, reliability, and selectivity.

### 4.1. Wild-Type AChE-Based Biosensors

#### 4.1.1. Immunosensors

**Figure 5:**
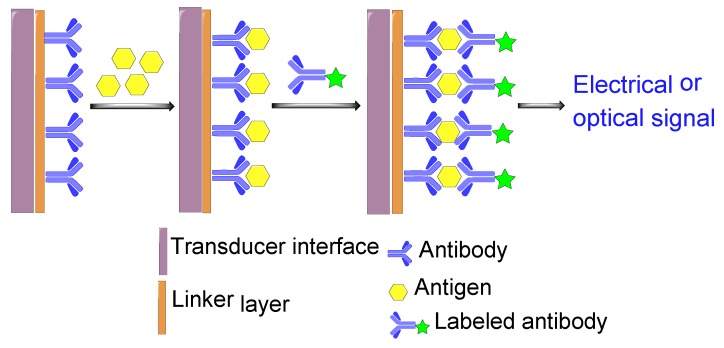
Schematic diagram of labeled antibody-based immunosensor.

Based on specific antigen-antibody interactions [42], immunosensors are favored for their high specificity, sensitivity, and stability. The advent of immunosensors has led to significant changes in traditional immunoassays. They integrate traditional immunoassay and biosensing technologies, and hence, combine many advantages. Immunosensors not only reduce analysis time, and improve sensitivity and testing accuracy, but also make the measurement process simple and easy to automate [43]. These types of sensors are classified into two types: labeled and non-labeled immunosensors. Figure 5 shows the schematic diagram of two steps involved in the labeled type of sandwich assay. The first step consists of the immobilization of the enzyme on a transducer surface to capture the corresponding analyte, while the second corresponds to the binding of a labeled secondary antibody with the captured analyte. Based on this principle, Sadik and Van Emon [44] reported in 1996 an immunosensing technology which forms immunocomplexes and produces a label signal that can be used to analyze environmental samples. Like most published AChE-based biosensors, this biosensor could only provide a global inhibition percentage induced by various toxic compounds present in the sample, drawing attention to the need for a differential biosensor. 

With this purpose, Bachmann and Schmid [45] constructed in 1999 an AChE-based multielectrode biosensor for the detection of paraoxon and carbofuran in mixtures. Four different AChEs (electric eel, bovine erythrocytes, rat brain, and *Drosophila melanogaster*) were immobilized by screen-printing on four-electrode thickfilm sensors in sets containing each AChE. The sensors monitored a detection range of 0.2–20 mg/l for both paraoxon and carbofuran within 60 min.

In 2008, Liu et al. [46] proposed a nanoparticle-based electrochemical immunosensor that was used to detect phosphorylated AChE. In this device, the former AChE was selectively adsorbed on zirconia nanoparticles (ZrO_2_ NPs) that were pre-coated onto a screen-printed electrode (SPE) by electrodeposition. Then, monoclonal anti-AChE was labeled with quantum dots (ZnS@CdS, QDs). The sandwich-like immunoreactions were formed with ZrO_2_ NPs, phosphorylated AChE, and ZnS@CdS, QDs. This immunosensor showed good performance because its voltammetric response was highly linear over the range of 10 pM to 4 nM of phosphorylated AChE, and the limit of detection (LOD) was estimated to be 8.0 pM. Similarly, Du et al. [47] reported an electrochemical immunosensor in 2011 that detects organophosphorylated acetylcholinesterase (OP-AChE), an exposure biomarker of organophosphate pesticides. ZrO_2_ NPs were immobilized on a screen-printed electrode (SPE) to capture OP-AChE complex via the interaction of metal chelation with phospho-moieties that were selectively identified using the lead phosphate-apoferritin labeled anti-AChE antibody (LPA–anti-AChE). The sensor forms a sandwich-like structure of ZrO_2_/OP-AChE/LPA–anti-AChE complex and detects lead ions on a disposable SPE.

In 2013, Zhao et al. [48] reported a photoelectrochemical (PEC) enzymatic biosensor whose system contained acetylcholine esterase (AChE) antibodies integrated with an ingenious photoelectrode. This photoelectrode was made of bismuth iodide oxide (BiOI) nanoflakes (NFs)/TiO_2_ nanoparticles (NPs) p–n heterojunction. In contrast to the majority of photoelectrochemical (PEC) enzymatic biosensors, this biosensor did not require enzyme immobilization on the surface, and could keep the initial catalytic ability of AChE with good sensitivity. These advantages facilitate the analysis of enzyme inhibition and activities. One year later, Ding et al. [49] designed an impedance immunosensor whose detection principle was based on the electrochemical characteristics of antigen-specific antibody immune response. They have developed the novel multilayer films based on Au nanoparticles (AuNPs) and polyaniline/carboxylated multiwall carbon nanotubes-chitosan nanocomposite (PANI/MWCNTs/CS). Compared with traditional methods, this immunosensor showed high speed and good consistency for the detection of pesticide residues.

#### 4.1.2. Nanomaterial-Based AChE Sensors

**Figure 6:**
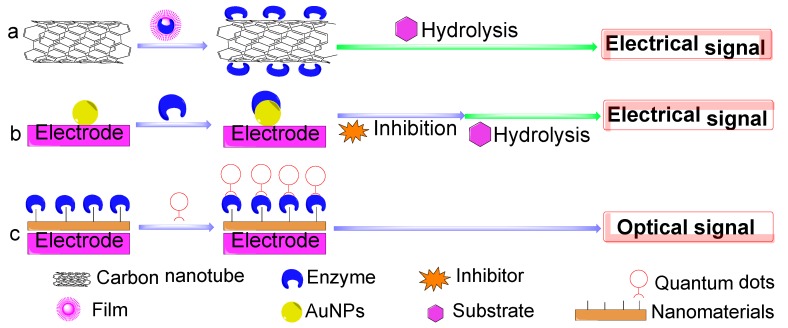
Schematic diagram of nanomaterial-based biosensors: (**a**) carbon nanotubes-based biosensor (**b**) Au nanoparticle-based electrochemical sensor (**c**) Quantum dots-based biosensor.

Nanomaterials were applied for modified electrodes to increase the conductivity and enzyme stability, and hence, the service life of sensor [50]. As depicted in Figure 6, the enzyme is usually immobilized on nanomaterials surfaces such as carbon nanotubes (CNTs), metal nanoparticles, and quantum dots [34]. The use of carbon nanotubes has a beneficial aspect, because they can be used at a low applied potential (+200 mV) without the use of redox mediator that reduces the electrochemical interferences [45]. 

In 2002, Pardo-Yissar et al. [51] reported a photoelectrochemical sensor based on AChE-labeled CdS nanoparticles that can be used for the detection of AChE inhibitors. The system contained AChE/CdS nanoparticles, an Au-electrode, and ACh. The hybrid system controls photocurrents by adjusting to the concentration of Ach, and is expected to be applied in different sensors.

In 2006, Liu and Lin [52] reported a flow injection amperometric biosensor with high sensitivity for which a cationic poly(diallyldimethyl ammonium chloride) (PDDA) layer and AChE layer formed the layer-by-layer (LBL) by electrostatic self-assembly. This electrostatic self-assembly is fixed onto negatively charged multiwalled carbon nanotubes (MWCNTs) modified by glassy carbon (GC) electrodes. The good bioactivity of AChE was due to the sandwich-like structure (PDDA/AChE/PDDA) on the CNT surface that provided a favorable microenvironment. Moreover, the electrocatalytic activity of CNT has greatly improved the electrochemical detection of the enzymatically generated thiocholine product owing to its low oxidation overvoltage (+150 mV), higher sensitivity, and increased stability. 

Based on the enzyme-induced growth of gold nanoparticles (AuNPs), Du et al. [53] developed in 2007 an AChE-based electrochemical sensor without adding gold nano-seeds. AChE was fixed onto an electrodeposited chitosan film with good biocompatibility. Three years later, Dan Du and co-workers [54] proposed another catalytic sensor for which AChE is fixed onto multi-walled carbon nanotubes (MWCNTs) incorporated with polypyrrole (PPy) and polyaniline (PANI) copolymer. The copolymer network provided a biocompatible microenvironment that increased the stability of the AChE biosensor. As an advantage, this sensor showed an increased capacity to promote electron transfer due to MWCNTs. The resulting AChE-based biosensor showed many advantages, such increased capacity to promote electron transfer, high sensitivity, good reproducibility, long-term stability, and low-cost processes, as well as being environment-friendly. However, the selectivity needs more improvement.

In 2014, Yang et al. [55] also described a nanohybrid-based electrochemical biosensor. More specifically, this biosensor (denoted as Au-PPy-rGO) was constructed by hybridizing gold nanoparticles, polypyrrole, and reduced graphene oxide sheets. AChE was co-deposited with (NH_4_)_2_SiF_6_ on the aforementioned matrix. The overall biosensor showed high stability and good binding affinity together with a fast response to OPs. Measurements taken under moderate conditions showed rapid and reliable detection of paraoxon-ethyl ranged from 1.0 nM to 5 μM with the LOD of about 0.5 nM.

In early 2018, Lu et al. [56] prepared a ultrasensitive electrochemical biosensors for the detection of OPs by integrating the newly designed Pd@Au core-shell nanorods with AChE. The developed biosensor exhibited higher sensitivity, reproducibility, and stability when sensing OPs in aqueous solution. For instance, it showed a linear relationship for the detection of paraoxon between of 3.6 pM and 100 nM. The LOD was calculated to be around 3.6 pM. In 2018, Jiang et al. [57] fabricated an acetylcholinesterase (AChE) biosensor AChE/Ag@Ti_3_C_2_Tx/GCE for electrochemical detection of malathion using Ag@Ti_3_C_2_Tx nanocomposites that improved the conductivity by enhancing the electron transfer via the enlargement of the specific surface area. The resultant AChE biosensor not only detected malathion in the linear range from 10^−14^ to 10^−8^ M, but also exhibited satisfactory selectivity, acceptable reproducibility, and good stability.

The application of Quantum Dots (QDs) to AChE-based biosensor is another field to which much focus has been directed over the past decade. As result, several papers have been published and recently reviewed by Zhou et al. [58]. On the basis of published literature, detection is based on the measurement of fluorescence (66.6%), current (11.1%), photocurrent (5.5%), phosphorescence (11.1%), and electrochemiluminescence (ECL) (5.5%) in this biosensor. The authors concluded that the use of QDs is beneficial compared with a conventional organic fluorophore for diverse reasons. For instance, QDs have adjustable fluorescence emission wavelength, broad absorption spectra, and the composition of inert inorganic with increased photochemical stability, intensive fluorescence, and longer fluorescence lifetime. These excellent optical properties make QDs an ideal fluorescent probe for the detection of OPs in various media. 

#### 4.1.3. Other Types of Sensors

Besides the aforementioned immune and nanomaterial-based sensors, other types of sensors have been reported for the detection of OPs. For example, in 2009, Zhang et al. [59] constructed a potentiometric enzymatic membrane biosensor for the detection of OPs. The principal component of this biosensor was a modified pH electrode. Indeed, an acetylcholinesterase layer formed by entrapment with *N,N*-dimethylformamide and bovine serum albumin was immobilized. With good agreement data obtained using a gas chromatography method, this biosensor showed a good linear signal with the detection limits of 10^–7^ mol/L for five pesticides (phorate, parathion, chlorpyrifos, methamidophos, and dimethoate). 

In 2006, Kim et al. [60] reported a quartz crystal microbalance (QCM) precipitation sensor. In this sensor, AChE was immobilized over the quartz crystal microbalance; its principle was based on the precipitation degree of an enzymatic reaction product, 3-indolyl acetate, that decreases in the presence of organophosphate EPN carbamate and carbofuran. Such biosensors significantly increased the sensitivity and binding efficiency with LODs of about 1.55 × 10^−8^ and 1.30 × 10^−9^ M, respectively. In the same year, Dale and Rebek [61] reported a small-molecule fluorescent sensor that can detect OP-containing nerve agents. The design rationale is very interesting. In general, the primary alcohol and a tertiary amine were introduced to mimic the key residues of AChE, while the fluorescent group was used as the optical readout. The proximity of OP nerve agent would trigger the subsequent reaction, leading to the fluorescence enhancement of the originally photoinduced electron transfer (PET) quenched sensor. 

In 2016, Tang et al. [62] developed another quartz crystal microbalance (QCM)-based acetylcholinesterase (AChE)-reduced graphene oxide (RGO) hybrid biosensor. This device, which could detect OP neurotoxin in the gas phase at room temperature, had improved sensibility, i.e., by about 8 times, compared to that of the AChE.

### 4.2. Wild-Type BChE-Based Biosensors

Although much effort has been directed to AChE, some BChE-based biosensors have been reported. For example, Makower et al. [63] described the possibility of detecting cholinesterase and its inhibitors such as diisopropylfluorophosphate (DFP) by a piezoelectric biosensor. Paraoxon was immobilized on the sensing surface as the recognition element, whereas BChE was used to detect the free organophosphates. The presence of free inhibitors prevented the binding of BChE to the surface-bound paraoxon due to the active site occupancy. The resultant biosensor exhibited a limit of detection of 10 nmol/L for DFP. Arduini et al. [25] constructed in 2007 a biosensor by immobilizing BChE onto screen-printed electrodes (SPE) modified with Prussian blue. Despite the notable lower inhibition rate of BChE, this device showed detection limits of 12 ppb against sarin standard solutions. More interestingly, it was able to detect the legal limit of sarin gas, which is 0.1 mg.m^−3^, within 30 s of incubation. 

Zhang et al. [19] reported in 2013 a Fe_3_O_4_ at TiO_2_ magnetic nanoparticles-based immunosensor for the detection of organophosphorylated BChE. The biosensor was labeled by quantum dots (QDs)-tagged anti-BChE antibody to amplify the electrochemical signal. The proposed immunosensor showed a linear response at OP-BChE concentrations ranging from 0.02 to 10 nM with a detection limit of 0.01 nM. Two years later, another BChE-based device was fabricated by Khaled et al. [26]. Based on the measurement of the inhibition percentage of BChE, the biosensor was able to determine ethion and its degradation products in the concentration range of 0 to 330 ng·mL^−1^. Recently, Sigolaeva et al. [27] constructed another BChE/microgel-based biosensor that showed a good long-term storage stability of 45 days at 4 °C and detection limits of 6 × 10^−12^ M and 8 × 10^−12^ M for diazinon (oxon) and chlorpyrifos(oxon), respectively after 20 min. This last example showed that, though not common, BChE-based biosensors could demonstrate improved properties. 

### 4.3. Biosensors Based on Cholinesterase Mutants

In some situations, mutants have great advantages, such as improved catalytic features. Protein engineering is generally used to get mutants with ideal properties that can overcome the limitation of wild-type enzymes [64]. Therefore, the main purpose of protein engineering is to increase the stability, sensitivity, and selectivity of the protein of interest [13]. This technology has been applied to engineered biosensors in which cholinesterase mutants were used to detect OPs.

As early as 1998, Villatte et al. [65] reported that the *Drosophila melanogaster* AChE mutant Y408F has great sensitivity to many organophosphates. In their study, the researchers first studied the sensitivity of AChE from different sources such as bovine erythrocyte, *Electrophorus electricus*, *Drosophila melanogaster*, *Torpedo californica* and *Caenorhabditis elegans*. As the wild-type *Drosophila melanogaster* AChE (*Dm*AChE) displayed the highest inhibition values, they then developed a *Dm*AChE mutant Y408F that increased the sensibility 12-fold for the detection of OPs traces. Moreover, the genetically modified *Dm*AChE are not denaturated in the presence of 5% acetonitrile, the solvent used to extract the pesticide from food samples [66]. From their study, it appeared that insect acetylcholinesterase was more sensitive to most organophosphates and carbamates pesticides.

Encouraged by the potential of *Dm*AChE mutant Y408F for the discovery of high sensitive ChE-based biosensor, Bachmann et al. [67] developed in 2000 another AChE-multisensor with improved selectivity for the detection of paraoxon and carbofuran in mixtures between 0 and 5 µg/L within 40 min. Compared with their earlier discovery [45], these improved properties were due to distinct variation in *ki* values of engineered AChE mutants, and the use of photosensitive PVA-SbQ as the enzyme immobilization matrix, which significantly increased the signals.

In 2005, Sofia Sotiropouloua et al. [68] reported a double mutant-based biosensor. Using site-directed mutagenesis, two mutations, E69Y and Y71D, were introduced in *Dm*AChE. More interestingly, this double mutated *Dm*AChE exhibited K_i_ value of 300 times higher than the wild-type *Dm*AChE. Encouraged by the improved activity of E69Y and Y71D mutants, they designed a biosensor for the detection of dichlorvos. The resultant sensor showed excellent sensitivity and selectivity with *k*_i_ of 487 μM^−1^·min^−1^, about 300- and 20,000-fold higher than those of wily type *Dm*AChE and *electrophorus electricus* AChE (*Ee*AChE), respectively. Moreover, the detection limit was decreased to 10^−17^ M, 5-fold lower than that of *Ee*AChE, due to the immobilization of the double mutant E69Y/Y71D to carbon nanomaterials. In the same year, Schulze et al. [69] constructed a sensitive biosensor by introducing mutations in *Nippostrongylus brasiliensis* AChE. This device could detect the maximum residue limit (10 μg/kg) in infant food of 11 out of the 14 most important OPs and carbamates with a detection limit of 3.5 × 10^−12^ M. More interestingly, the biosensor showed high storage stability for 17 months. This example shows that, besides *Drosophila melanogaster,* whose ChE is known to be the most sensitive towards OPs, other species could also give significant promising results.

A sensitive amperometric biosensor for the quantification of dichlorovos in apple skin was also fabricated using modified AChE [70]. This biosensor showed an improved detection limit of 7 × 10^−11^ M and 1 × 10^−8^ M for mutant and wild-type, respectively. Mishra et al. [71] presented in 2015 an automatic flow based biosensor that was used to detect chlorpyriphos-oxon and malaoxon OPs mixtures in milk. Based on artificial neural network (ANN) algorithm, genetically modified acetylcholinesterase (AChE) were immobilized on screen-printed electrodes (SPEs) and used as bioreceptors. The resultant sensor showed detections limits ranging from 5 × 10^−10^ to 5 × 10^−12^ M and 1.01 × 10^−10^ to 9.17 × 10^−11^ M for chlorpyriphos-oxon and malaoxon, respectively. For the ease of comparison, some critical parameters, such as the limit of detection (LOD), the detection, and lifetime of selected recent ChE-based biosensors are shown in Table 1.

## 5. Conclusions

Engineered cholinesterases are reported to have potential for the detection of OP residues from environmental samples and food. Among many methods that have been reported, sensors are a powerful analysis method for the fast detection of OPs. Recourse to the use of ChE-based biosensors for detecting OPs and carbamates, the most concerning pesticides, is a promising research field. Thus, many ChE-based biosensor models have been proposed with improved properties including sensitivity, processing speed, and specificity [41]. These improvements were due to the use of genetically modified ChEs and advanced nanoscience and nanotechnology, that promise a better future for designing of AChE-based biosensors. However, despite extensive research and promising improvements, few ChE biosensors are used in real applications due to some limitations that need to be addressed. 

These limitations include the low enzyme storage stability, and susceptibility to reactions with inhibitors [72,73,74,75]. Apart from biosensors used for the detection of carbamates that can be reactivated [76], a lack of repetitive use of the same biosensor without enzyme reloading may increase the related cost. Also, except for some biosensors that could discriminate between two analytes [28,45,67], most reported bioesensors can only detect single analytes in mixtures of OPs, calling for more research, given the number of OPs present in the same sample. It is worth mentioning that if these limitations are not addressed, esterase-based biosensors will represent a viable alternative to automated robotic systems in achieving continuous monitoring for risk assessment associated with pesticides [28].

ChE-based biosensors are believed to provide good performance for the detection of OPs and carbamante residues. In the future, compact, portable, sensitive, reliable, selective, and long-lasting, automated devices which are specifically designed for real applications will constitute an intensive research area for ChE-based biosensors. Significant progress is expected in genetic engineering in combination nanoscience and nanotechnology. 

## Figures and Tables

**Table 1:** Comparison of selected recent ChE-based biosensors with improved limit of detection (LOD).

Bioreceptor	LOD (M)	Analyte	Detection Time (min)	Lifetime (days)	Reference
*Dm*AChE	7.26 × 10^−8^ 9.03 × 10^−8^	paraoxoncarbofuran	60 nd	nd nd	[45]
humanAChE/ZrO_2_ NPs	8 × 10^−9^	human plasma	nd	nd	[46]
humanAChE/ZrO_2_ NPs	2 × 10^−11^	paraoxon	nd	nd	[47]
horseAChE/ZrO_2_ NPs	5.36 × 10^−7^ 2.35 × 10^−8^	carbaryl dichlorvos	12 nd	21 nd	[49]
AChE */CNT	4 × 10^−13^	paraoxon	6	7	[52]
AChE */AuNPs	9.08 × 10^−11^	malathion	nd	nd	[53]
*Ee*AChE/MWCNTs	3.02 × 10^−9^	malathion	nd	nd	[54]
*Ee*AChE	5 × 10^−10^	paraoxon-ethyl	nd	30	[55]
AChE */Pd@Au NRs	3.6 × 10^−12^	paraoxon	nd	30	[56]
*Ee*AChE/Ag@Ti_3_C_2_Tx	3.27×10^−15^	malathion	nd	7	[57]
*Nb*AChE mutant	3.5 × 10^−12^	pirimiphos methy	30	257	[69]
*Dm*AChE	1.59 × 10^−9^ 1.81 × 10^−9^	malaoxon paraoxon	40 40	nd nd	[67]
*Dm*AChE mutant	10^−17^	dichlorovos	10	nd	[68]

* the source is not specified; *Dm*AChE, *Drosophila melanogaster* AChE; EeAChE, electric eel AChE; *Nb*AChE, *Nippostrongylus brasiliensis* AChE.
